# Welcome to the Family: Identification of the NAD^+^ Transporter of Animal Mitochondria as Member of the Solute Carrier Family SLC25

**DOI:** 10.3390/biom11060880

**Published:** 2021-06-14

**Authors:** Mathias Ziegler, Magnus Monné, Andrey Nikiforov, Gennaro Agrimi, Ines Heiland, Ferdinando Palmieri

**Affiliations:** 1 Department of Biomedicine, University of Bergen, 5009 Bergen, Norway; 2 Department of Biosciences, Biotechnologies and Biopharmaceutics, University of Bari, Via E. Orabona 4, 70125 Bari, Italy; gennaro.agrimi@uniba.it (G.A.); ferdpalmieri@gmail.com (F.P.); 3 Department of Sciences, University of Basilicata, Via Ateneo Lucano 10, 85100 Potenza, Italy; 4 Institute of Cytology, Russian Academy of Sciences, 194064 St. Petersburg, Russia; andrey.nikiforov@gmail.com; 5 Center of Excellence in Comparative Genomics, University of Bari, Via E. Orabona 4, 70125 Bari, Italy; 6 Department of Arctic and Marine Biology, UiT The Arctic University of Norway, 9037 Tromsø, Norway; ines.heiland@uit.no; 7 Department of Clinical Medicine, University of Bergen, 5009 Bergen, Norway

**Keywords:** mitochondrial carrier, mitochondrial transporter, membrane transport, mitochondria, solute carrier family 25, SLC25, SLC25A51, NAD^+^ transporters, NAD

## Abstract

Subcellular compartmentation is a fundamental property of eukaryotic cells. Communication and metabolic and regulatory interconnectivity between organelles require that solutes can be transported across their surrounding membranes. Indeed, in mammals, there are hundreds of genes encoding solute carriers (SLCs) which mediate the selective transport of molecules such as nucleotides, amino acids, and sugars across biological membranes. Research over many years has identified the localization and preferred substrates of a large variety of SLCs. Of particular interest has been the SLC25 family, which includes carriers embedded in the inner membrane of mitochondria to secure the supply of these organelles with major metabolic intermediates and coenzymes. The substrate specificity of many of these carriers has been established in the past. However, the route by which animal mitochondria are supplied with NAD^+^ had long remained obscure. Only just recently, the existence of a human mitochondrial NAD^+^ carrier was firmly established. With the realization that SLC25A51 (or MCART1) represents the major mitochondrial NAD^+^ carrier in mammals, a long-standing mystery in NAD^+^ biology has been resolved. Here, we summarize the functional importance and structural features of this carrier as well as the key observations leading to its discovery.

## 1. Introduction

Transmembrane transport proteins (transporters or carriers) catalyse the translocation of specific ions, nutrients, metabolites, cofactors, and proteins across biological membranes, which otherwise would be impermeable to these molecules. It has been estimated that 10% of all human genes encode polypeptides involved in solute transport across the plasma and intracellular membranes [1]. Based on sequence homology, more than 400 human transporters have been classified into 66 solute carrier (SLC) protein families, excluding active transporters (ABC transporters and ATPase pumps) and ion channels [1,2]. For example, members of the major facilitator superfamily, which have twelve transmembrane helices, are subdivided into various SLC families, and mitochondrial carriers, which have six transmembrane helices, belong to the SLC25 family. About 30% of the SLCs are orphans, i.e., what they transport is unknown. There is also a long list of molecules that are probable substrates of yet unidentified transporters based on the knowledge about the cellular compartmentalization of enzymes and biochemical compounds. It is a great challenge to find the missing pieces in this metabolic puzzle, trying to identify the physiological substrate for a transporter or vice versa.

**Figure 1:**
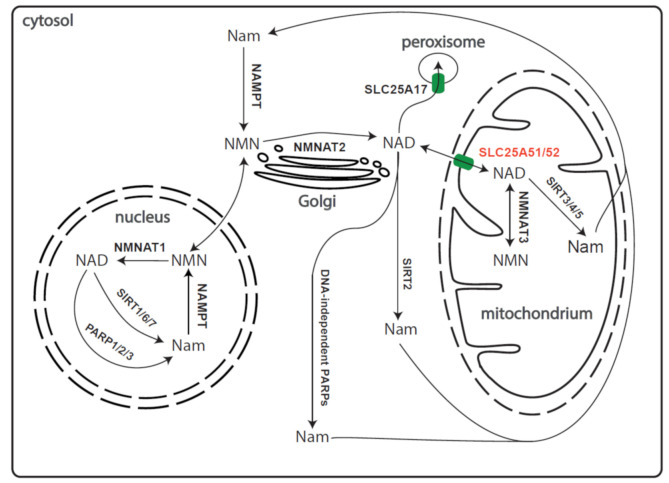
Compartmentalisation of NAD^+^ biosynthesis and salvage pathway in human cells. The figure shows the compartmentalisation of NAD^+^ synthesis, consumption, and salvage. The three different isoforms of NMNAT show distinct subcellular localisations with NMNAT1 primarily localized to the nucleus, NMNAT2 localized to the Golgi apparatus, and NMNAT3 in the mitochondria. The peroxisomal and mitochondrial SLC transporters are highlighted in green. (Abbrev.: Nam: nicotinamide; NMN: nicotinamide mononucleotide; NMNAT1/2/3: NMN adenylyltransferase 1/2/3; SIRT: sirtuin; PARP: poly-ADP-ribosyl polymerase; NAMPT: Nam phosphoribosyltransferase).

Nicotinamide adenine dinucleotide (NAD^+^) is the most widely used cofactor of enzymatic redox reactions in the cell. It has the capacity to be an electron donor in its reduced form (NADH) and electron acceptor in its oxidized form (NAD^+^). It is also an important signalling molecule involved in a multitude of signalling processes in different cellular compartments. NAD^+^ appears to be present in all cellular organelles, with mitochondria containing up to 70% of the total cellular NAD^+^ [3]. There are several routes of NAD^+^ biosynthesis. With regard to the pyridine base, NAD^+^ can be synthesized from several different precursors in animals: nicotinamide (Nam) and nicotinic acid (together known as vitamin B3), tryptophan, and nicotinamide riboside (NR). They are obtained from diet and imported into cells by various SLC transporters (SLC5A8, SLC22A13, and members of the SLC29 family for vitamin B3; e.g., SLC7A5 and SLC36A4 for tryptophan) [4,5]. The majority of NAD^+^ is synthesized from nicotinamide, which is also released by NAD^+^ consuming signalling reactions (Figure 1). The first enzyme of the so-called NAD^+^ salvage pathway is nicotinamide phosphoribosyl transferase (NAMPT) and is predominantly localized to the cytosol and the nucleus [6,7]. To maintain mitochondrial NAD^+^ concentrations, cytoplasmic NAD^+^ or an NAD^+^ intermediate, such as nicotinamide mononucleotide (NMN), need to be imported into mitochondria. It has been known for a long time that NADH cannot be transported directly into mitochondria. Various systems have evolved to shuttle the reducing equivalents of cytosolic NADH into the mitochondrial matrix, e.g., the malate-aspartate and glycerol-3-phosphate shuttles [8,9]. Proteins, which all belong to the mitochondrial carrier family, have been identified that transport NAD^+^, and to a lesser extent, NMN and nicotinic acid adenine dinucleotide (NAAD), in yeast and plant mitochondria [10,11] or NAD^+^ into human and plant peroxisomes [12,13,14]. However, until very recently, no NAD^+^ transporter had been found in animal mitochondria.

In 2020, SLC25A51 (along with SLC25A52, which is 96% identical) was concluded to be a human mitochondrial NAD^+^ transporter by three independent research teams [15,16,17]. Although SLC25A51 is a member of the mitochondrial carrier family, it is not a close homologue of previously identified NAD^+^ carriers. Using functional assays, yeast complementation, and genetic engineering, the role of SCL25A51 as a human mitochondrial NAD^+^ transporter was established. These approaches are different from the EPRA method (where the protein is recombinantly expressed, purified, and reconstituted into liposomes for transport assays in vitro) that has been applied for the identification of the NAD^+^ carriers in plants and yeast and the majority of the other SLC25 members. The SLC25 carrier family transports a wide range of substrates from protons, inorganic ions, and small metabolites to nucleotides and large cofactors [18]. In this review, human NAD^+^ metabolism and its compartmentalisation as well as the discovery and characteristics of SLC25A51 and the other NAD^+^ carriers are discussed.

## 2. Human NAD^+^ Biosynthesis and Its Compartmentation

In human cells, the majority of NAD^+^ is produced from Nam in a two-step pathway. The first step is catalysed by NAMPT in which NMN is formed from Nam and phosphoribosylpyrophosphate (PRPP). The affinity of NAMPT towards its substrates is enhanced by autophosphorylation of the enzyme, ATP being the phosphoryl donor [19]. Under these conditions, the affinity of NAMPT towards Nam is in the low nanomolar range, thereby assuring efficient recycling of Nam produced from NAD^+^ in signalling reactions (Figure 1) [20]. Formation of the dinucleotide, NAD^+^, is accomplished by NMN adenylyltransferases, NMNATs. Mammalian cells have three NMNAT genes encoding isoforms that are present in the nucleus (NMNAT1), the Golgi complex, facing the cytosol (NMNAT2) and the mitochondria (NMNAT3) (Figure 1).

In addition to the NAMPT-dependent NAD^+^ synthesizing pathway, alternative precursors can also maintain cellular NAD^+^ homeostasis. Quinolinic acid, a product of tryptophan degradation in the kynurenine pathway, is converted to NAMN, the acidic form of NMN, and thereby enters NAD^+^ synthesis [21]. This route appears to be important for liver NAD^+^ homeostasis, at least in mice [22]. Likewise, nicotinic acid is also converted to NAMN by nicotinic acid phosphoribosyltransferase. NMNATs form the dinucleotide, NAAD, from NAMN and ATP, in the same manner as NAD^+^ from NMN and ATP [21]. Thus, all NAD^+^ biosynthetic pathways require NMNAT activity [23]. In mammals, NAAD is amidated to NAD^+^ by NAD^+^ synthetase using glutamine as an amide donor and the energy of ATP to accomplish the reaction [21]. Thereby, the conversion of Nam to NAD^+^ through NAMPT activity is not only the shortest, but also the energetically least-demanding pathway. This pathway is also known as Nam salvage pathway as it is required to resynthesize NAD^+^ from Nam, generated by NAD-consuming signalling reactions. NAD-dependent signalling reactions such as the de-acetylation of proteins by sirtuins or the mono- and poly-ADP-ribosylation through ADP-ribosyl transferases (ART) or poly-ADP-ribosyltransferases (PARPs) [4,24,25], have been shown to lead to a constant turnover of cellular NAD^+^ pools, resulting in half-lives of only a few hours in human cells [22].

In recent years, the use of the nucleoside of Nam, NR, has gained tremendous interest. Having the ribose already attached, it can be readily converted to NMN by phosphorylation, thereby providing an efficient, PRPP-independent route to NAD^+^ [25,26]. Since NAMPT represents the rate-limiting reaction in human NAD^+^ biosynthesis, bypassing it using NR can potentially boost cellular NAD^+^ levels with a wide range of beneficial physiological effects. Likewise, dietary supplementation with NMN has also been proposed as a strategy to improve NAD^+^ availability [25,27]. Physiological improvements have been largely ascribed to the activation of NAD-dependent signalling pathways, in particular, SIRT-dependent protein deacetylation. Furthermore, some beneficial effects of NAD^+^ supplementation have been associated with enhanced mitochondrial functions, mediated, at least in part, by the activation of nuclear SIRT1, resulting in transcriptional activation of genes promoting mitochondrial proliferation. However, there also appear to be direct mitochondrial effects that might be explained by increased availability of NAD^+^ within these organelles. It should be noted that, so far, effects of NR supplementation on mitochondrial function have largely been observed in preclinical animal models, not humans.

NAD-dependent metabolic and signalling processes are strictly compartmentalized. This raises the question how various NAD^+^ pools are formed and maintained, and how they interact. To date, the cytosolic, nuclear, and mitochondrial NAD^+^ pools have been best described. Using the PARAPLAY assay, which is based on expression of the catalytic domain of poly-ADP-ribosyltransferase 1 (PARP1) in various subcellular compartments [28], pools of NAD^+^ in peroxisomes, ER, and Golgi complex [29,30] were demonstrated. It was shown that NAD^+^ is transported into peroxisomes using the mitochondrial type carrier SLC25A17 [13]. How NAD^+^ enters the ER or the Golgi complex is currently unknown.

For a long time, nuclear and cytosolic NAD^+^ were thought to form a single pool as it was believed that the free dinucleotide can diffuse through nuclear pores whose diameters are much larger than the size of NAD^+^. The analysis is complicated by the fact that both NAMPT and NMNAT activities are present in both compartments and recent work has demonstrated that the interactions between the nuclear and cytosolic NAD^+^ pools are complex. Using genetically encoded biosensors for NAD^+^ targeted to various cellular compartments, it was suggested that the depletion of NMNAT2 in HEK293T cells decreased cytoplasmic but not nuclear NAD^+^ concentrations [31]. Ryu et al. [32] found that stimulation of NAD^+^ synthesis in the cytosol, through the induction of NMNAT2, leads to a decrease in the level of NAD^+^ in the nucleus and suppression of PARP1 activity, regulating the expression of genes involved in adipogenic differentiation. Moreover, a model was proposed according to which the NAD^+^ nuclear pool is regulated by competition between NMNAT1 and NMNAT2 for their common substrate, namely NMN. Thus, despite the fact that we cannot exclude the direct exchange of NAD^+^ between the cytosol and the nucleus, depletion of the dinucleotide in one compartment cannot be fully offset by another pool. This may result in the nonviability of embryos observed in mice knocked out for NMNAT1 [33] or NMNAT2 [34].

## 3. The Mitochondrial NAD^+^ Pool

Despite the important role of the mitochondrial NAD^+^ pool for cellular metabolism and regulation, there are still many open questions related to its formation and maintenance. As most available evidence suggests NMNAT3 to be a mitochondrial isoform [6,35], it has been thought that NMNAT3 is essential to establish and maintain the mitochondrial NAD^+^ pool. It has been shown that an increase in the level of cytosolic NMN leads to an increase in the level of mitochondrial NAD^+^ in cultured human cells [6]. Based on these data, it was suggested that the cytosolic precursor of mitochondrial NAD^+^ is the mononucleotide NMN, which, after being imported into the mitochondrial matrix, is adenylated to the dinucleotide NAD^+^ by NMNAT3. This hypothesis was also supported by in vitro data, according to which mitochondria isolated from rat liver can synthesize NAD^+^ from NMN and ATP [36]. Using mitochondria isolated from murine skeletal muscle or C2C12 myoblasts, Davila et al. [37] confirmed this observation. However, they demonstrated that the NMNAT activity was not located in the mitochondrial matrix but depended on the nuclear enzyme NMNAT1 [37]. Consequently, the detected mitochondria-associated NMNAT activity may have been the result of a contamination with the nuclear protein. Increased expression of NMNAT3 in transgenic mice and in cultured cells, however, efficiently increases mitochondrial NAD^+^ levels in various tissues [38,39]. Suppressed expression of this protein in some cells leads to a significant decrease in mitochondrial NAD^+^ concentrations, while in others, it has no effect on the mitochondrial NAD^+^ pool [31]. Mice lacking Nmnat3 have, nevertheless, normal mitochondrial dinucleotide levels in most tissues [40]. Moreover, NMNAT3 was found to play a key role in maintaining the NAD^+^ pool in mature erythrocytes in which mitochondria are absent [41].

Thus, data on the role of NMNAT3 in maintaining the mitochondrial NAD^+^ pool have remained controversial. NMNAT3 may be an important regulator of mitochondrial NAD^+^ content, but there need to be alternative mechanisms that mediate the generation of the mitochondrial NAD^+^ pool.

In recent years, the focus of studies on the mechanisms of maintenance of mitochondrial NAD^+^ in mammals has shifted to reconsider the possibility of a transporter carrying the dinucleotide into the organelles. It was found that NMNAT2 depletion in HEK293T and HeLa cells decreased cytoplasmic and mitochondrial NAD^+^ levels, whereas NR did not restore mitochondrial NAD^+^ concentrations in NMNAT2 knockdowns in HeLa cells. These data suggested that NAD^+^ produced in the cytoplasm can influence mitochondrial stores [31]. Moreover, using C2C12 cells and NAR labelled with two different isotopes for ribose and Nam (13C on the pyridine carboxyl group and a deuterium on the ribose moiety), Davila et al. [37] demonstrated the incorporation of both labels into mitochondrial NAD^+^. Furthermore, given that NAR is converted to NAD^+^ in the cytosol via NAMN and NAAD, the cytosolic NMN pool was not labelled. This led to the suggestion that the mitochondrial NAD^+^ pool can be established through direct import of NAD^+^ [37]. These observations prompted intensive research efforts in various laboratories to address the possibility of the existence of a mitochondrial NAD^+^ carrier in mammalian cells.

## 4. Identification of Mitochondrial NAD^+^ Transporters in Yeast, Plants, and Bacteria

The mitochondrial NAD^+^ carriers in *Saccharomyces cerevisiae*, Ndt1p, and Ndt2p, which have 70% sequence identity, belong to the mitochondrial carrier family having the typical signature motif sequences (conserved consensus sequences initiating with PX[DE]XX[KR]) in three tandemly repeated domains [10]. These two proteins were addressed because they cluster with mitochondrial nucleotide carriers in phylogenetic trees and especially close to the FAD transporter Flx1p and pyrimidine nucleotide transporter Rim2p, which both have a rare tryptophan instead of the first negatively charged residue of the second signature motif just like Ndt1p and Ndt2p [10]. The substrates of Ndt1p were identified by the EPRA method, i.e., by expression in *Escherichia coli*, purification, reconstituted into liposomes and transport assays [10,18,42]. Ndt1p, which was found to be localized to mitochondria as well as Ndt2p, mainly transports NAD^+^ (Km of 0.38 mM), and to a lesser extent, (d)AMP, (d)GMP, and NAAD. In addition, the nucleotides of uridine, thymine, and cytosine were transported by Ndt1p less efficiently than those of adenine and guanine with the following order of potency: monophosphates > diphosphates > triphosphates. By contrast, α-NAD^+^, NADH, NMN, NAMN, as well as FAD and FMN are very poor substrates of Ndt1p; and NADP, NADPH, Nam, and nicotinic acid are not transported at all by Ndt1p [10]. Furthermore, this carrier functions as a slower uniporter and a faster antiporter of substrates. In addition, yeast cells lacking Ndt1p or Ndt2p have growth defects on non-fermentable carbon sources and reduced mitochondrial levels of NAD^+^, a phenotype that becomes more pronounced in double knockout cells [10,43]. It should be emphasized that in *S. cerevisiae*, there is good evidence that there are no NAD^+^ biosynthetic enzymes within mitochondria [10]. Based on the biochemical characterization of Ndt1p, the high sequence identity between Ndt1p and Ndt2p, and the properties of the deleted strains, it was concluded that the main physiological role of both Ndt1p and Ndt2p is to import NAD^+^ from the cytosol into the mitochondrial matrix in exchange for (d)AMP and (d)GMP [10].

Two of the 58 mitochondrial carriers of *Arabidopsis thaliana*, AtNDT1 and AtNDT2 (with 61% sequence identity in their carrier domains), display considerable similarity to yeast Ndt1p and Ndt2p with percentages of identity ranging from 26 to 34% [11]. The transport properties of these two plant carriers were characterized by the EPRA method, demonstrating that NAD^+^ is transported by both of them (Km values in submillimolar range) as well as NAAD, NMN, ADP, and AMP, and to a lesser extent NAMN, FAD, FMN, and several other nucleotides with the bases A, G, C, U, and T, whereas pyrophosphate is only transported by AtNDT1 [11]. The specific activity of AtNDT2 is about three times higher than that of AtNDT1 and both carriers exhibit antiport transport rates much higher than those of uniport. Furthermore, the expression of AtNDT1 and AtNDT2 in yeast lacking Ndt1p and Ndt2p restores mitochondrial NAD^+^ transport. Initially, subcellular localization studies of AtNDT1 and AtNDT2 suggested that they are found in chloroplasts and mitochondria, respectively [11]. However, later, AtNDT1 was reported to localize to mitochondria [44], in agreement with previous unpublished results (E. Blanco and F. Palmieri, personal communication). Both proteins are expressed in developing and metabolically active tissues: AtNDT1 in leaf mesophyll cells and root tips; AtNDT2 in meristematic shoots, vascular bundles of leaves, siliques, petal veins, pollen, and roots [11]. Diminished expression of AtNDT1 in *Arabidopsis* leads to impairments in the reproduction system, such as reduction in pollen, silique length, and seeds, whereas the vegetative growth is increased due to enhanced photosynthesis, leaf number, starch, and sucrose levels [44]. Reduced expression of AtNDT2 also affects the plant reproductive phase, in this case by decreasing seed quantity and quality [45]. Moreover, reduced AtNDT2 levels in flowers and seedlings trigger increased expression of several enzymes involved in NAD^+^ biosynthesis. In conclusion, AtNDT1 and AtNDT2 catalyse the import of NAD^+^ into mitochondria, probably in exchange for intramitochondrial ADP or AMP.

Two other members of the mitochondrial carrier family have been found to transport NAD^+^, the human SLC25A17 and the *Arabidopsis* PXN. Notably, at variance to all the other NAD^+^-transporting proteins mentioned above, these two carriers are localized to peroxisomes. Both of them have been characterized biochemically by the EPRA method. Although SLC25A17 transports NAD^+^ along with PAP (adenosine 3′,5′-diphosphate) and ADP to a lesser extent than the main substrates of this carrier (CoA, FAD, FMN, and AMP), it is most likely that SLC25A17 also plays a role in peroxisomal NAD^+^ import in humans [13]. PXN transports NAD^+^ (Km values in submillimolar range), NADH, AMP, and ADP at low rates, in addition to CoA, dephospho-CoA, PAP, and acetyl-CoA that are transported at higher rates [12,14], and is therefore also involved in peroxisomal NAD^+^ import in plants.

It should be mentioned that membrane transporters not belonging to the mitochondrial carrier family have also been identified to transport NAD^+^: the plasma membrane nucleotide transporter NTT4 and Npt1*_Ct_* of *Protochlamydia amoebophila* UWE25 and *Chlamydia trachomatis*, respectively. Both are obligate intracellular symbionts in protozoa [46,47]. These two transporters are of the NTT family, whose members are found in intracellular bacteria and plant plastids and are predicted to have 10–12 transmembrane helices.

## 5. Discovery of a Mammalian Mitochondrial NAD^+^ Carrier

It has long been believed that in human and animal cells, there is no transport of NAD^+^ between mitochondria and the cytosol. This idea was supported in in vitro experiments with mitochondria isolated from rat liver, in which it was shown that NAD^+^ does not pass through the inner membrane of the organelle [36]. The absence of NAD^+^ transport between the cytosol and mitochondria was also supported by data on the maintenance of mitochondrial NAD^+^ within physiological concentrations with significant depletion of other pools (cytosolic and nuclear) evoked by genotoxic stress during treatment of cells with the DNA alkylating agent MMS [48] or after suppression of the activity of the NAMPT by the inhibitor FK866 [49]. The only alternative to the import of NAD^+^ from the cytosol seemed to be intramitochondrial synthesis.

On the other hand, mitochondrial NAD^+^ carriers have been identified in yeast and plants [10,18]. Previous attempts to detect a mitochondrial NAD^+^ transporter in human cells based on homology with the characterized transporters in yeast (Ndt1p and Ndt2p) and plants (AtNDT1 and AtNDT2) were unsuccessful. It was shown that the mitochondrial folate and FAD carrier SLC25A32, which is the closest homologue in mammals of the above-mentioned characterized NAD^+^ transporters, does not influence mitochondrial NAD^+^ [35,48]. Other potential candidates from the SLC25 family–the pyrimidine nucleotide transporters SLC25A33 and SLC25A36—did not exhibit NAD^+^ transporter activity either [35,50]. Consequently, alternative mechanisms of mitochondrial NAD^+^ generation have been forwarded; in particular, those including mitochondrial NMNAT3 [6,35]. However, more recent research showed, albeit indirectly, that mitochondrial NAD^+^ originates from the cytosol [37]. Eventually, at the end of 2020, three research groups independently characterized the mitochondrial NAD^+^ transporter in human cells-SLC25A51 (also called MCART1) [15,16,17].

Genetic coessentiality analyses showed strong negative interactions of SLC25A51 with mitochondrial transporters essential for one-carbon and glutathione metabolism as well as a strong negative interaction with the glucose transporter at the plasma membrane *SLC2A1*/GLUT1. Since SLC25A51, furthermore, showed negative interactions with mitochondrial transporters for other cofactors, it was a likely candidate for the mitochondrial NAD^+^ transporter. Targeted metabolomics in SLC25A51-deficient cells showed a depletion of riboflavin as well as purine nucleotides and an increased level of glutathione metabolism-related metabolites [15,16]. The role of SLC25A51 as a NAD^+^ transporter was further confirmed through knockouts in various cell lines, which led to dramatic impairment of mitochondrial respiration [15,16,17].

In a variety of cells, knockout or knockdown of the gene encoding the SLC25A51 protein resulted in a decreased oxygen consumption rate because of impaired respiratory complex I activity [15,16,17]. LC-MS-based metabolomics analyses of SLC25A51 KO cells revealed extensive alterations in major mitochondrial metabolic pathways such as the TCA cycle, one-carbon flux, fatty acid oxidation, and ETC function [15,16]. Moreover, cells lacking SLC25A51, cultivated in a medium containing galactose instead of glucose as a carbon source, were unable to generate ATP in mitochondria [16]. Thus, SLC25A51 deficiency led to dramatic impairment of mitochondrial respiration. Using targeted metabolomics, a genetically encoded cpVenus NAD^+^ sensor or the PARAPLAY system, it was shown that SLC25A51 deficiency selectively depleted mitochondrial (but not whole cell) NAD^+^, whereas overexpression of SLC25A51 significantly increased mitochondrial NAD^+^ levels [15,16,17]. It was also found that NAD^+^ uptake into mitochondria isolated from SLC25A51 KD or KO cells was substantially decreased compared to control mitochondria. On the contrary, mitochondria from cells overexpressing SLC25A51 demonstrated increased NAD^+^ uptake capacity [16,17]. It is noteworthy that in in vitro experiments using 13C5-NAD^+^, unlabelled NAD^+^ or NADH, but not NMN, competed for isotope-labelled NAD^+^ uptake into mitochondria [16]. Moreover, to validate the function of SLC25A51 as NAD^+^ transporter, it was tested whether the human SLC25A51 and yeast mitochondrial NAD^+^ transporters Ndt1p and Ndt2p functionally substitute each other. Overexpression of the SLC25A51 protein rescued slow growth and normalized mitochondrial NAD^+^ levels of the yeast strain in which both *NDT1* and *NDT2* were deleted [15,16]. Ectopically expressed SLC25A51 also fully rescued impaired uptake of 3H-NAD^+^ into mitochondria isolated from NDT1 and NDT2 double knockout yeast [17]. Using this experimental system, the authors also determined kinetic parameters of SLC25A51-mediated NAD^+^ transport (Km = 200 μM ± 60 μM; V*max* = 1200 pmol sec^−1^ mg^−1^) On the other hand, overexpression of the yeast protein Ndt1p rescued mitochondrial NAD^+^ levels and mitochondrial respiration in human SLC25A51 knock out cells [15,16]. Thus, it was convincingly demonstrated that the proteins SLC25A51 and Ndt1p (as well as Ndt2p) are functional homologues.

Despite the fact that the mitochondrial NAD^+^ uptake was not confirmed by a direct transport method upon reconstitution of the protein into liposomes, it is highly probable that SLC25A51 is indeed a mitochondrial NAD^+^ carrier in human cells. Further validation and investigation will provide more insights into the transport characteristics and the physiological effects of alterations in SLC25A51 expression.

Available data show that SLC25A51 expression is relatively constant across human tissues, with highest expression in testis, breast, bone marrow, parathyroid gland, and adipose tissues. Notably, expression of the close homologue SLC25A52 is mainly detectable in testis with some low-level expression in other tissues [51].

## 6. Molecular Characterisation of the Mitochondrial Carrier SLC25A51

**Figure 2:**
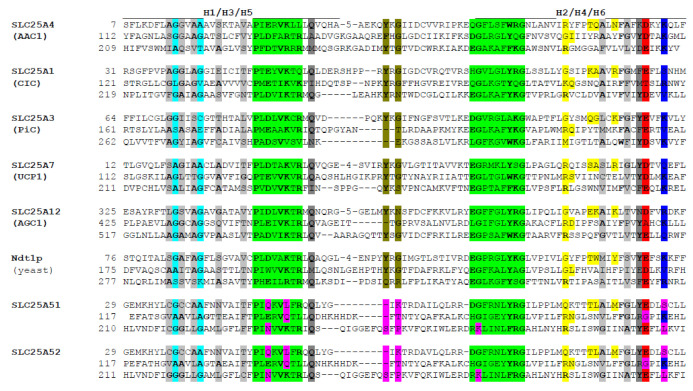
Sequence comparison of the three mitochondrial carrier domain repeats in nucleotide transporter SLC25A4 (ADP/ATP carrier 1, AAC1), carboxylate transporter SLC25A1 (citrate carrier, CIC), phosphate transporter SLC25A3 (PiC), proton transporter SLC25A7 (uncoupling protein 1, UCP1), amino acid transporter SLC25A12 (aspartate-glutamate carrier 1, AGC1), NAD^+^ transporter Ndt1p (*S. cerevisiae*), SLC25A51, and SLC25A52. The following sequence features are indicated: position of the transmembrane helices H1-6 (a line above the sequences), the conserved residues (bold), the helical packing GXXXG motif (cyan), other helical packing small amino acids (light grey), the signature motif sequences (green), the glutamine and tyrosine braces (dark grey), the cardiolipin-binding motif 1 (olive), the contact point residues of the proposed substrate binding site (yellow), and the negatively (red) and positively (blue) charged residues of the cytosolic gate. The distinct sequence variations in SLC25A51 (and SLC25A52) discussed in the text are indicated in magenta.

SLC25A51 has been included into the solute carrier family 25 (SLC25), also named mitochondrial carrier family (MCF), due to the presence of three signature motif sequences in three tandemly repeated 100-residue domains, which are characteristic for the members of this protein family (Figure 2) [52,53,54,55]. In fact, SLC25A51 was initially called Mitochondrial Carrier Triple Repeat Protein 1 (MCART1). Typically, each 100-residue domain of a mitochondrial carrier (MC) contains two transmembrane helices and the conserved residues of the signature motif (PX[DE]XX[KR]X[KR]X_20–30_[DE]GXXXX[WYF][KR]G; PROSITE PS50920, PFAM PF00153, and IPR00193). The first part of the signature motif (PX[DE]XX[KR]X[KR) marks the end of the odd-numbered transmembrane helices, whereas the last residues are located just before the beginning of the even-numbered transmembrane helices. In humans, the SLC25 family consists of 53 members annotated from SLC25A1 to SLC25A53 and many of them have been found to transport nucleotides, amino acids, carboxylates, inorganic anions, and cofactors across the inner mitochondrial membrane [18,56,57,58], and mutations in 18 of these MCs cause human diseases [59]. The six transmembrane helices (H1–H6) of MCs are structured in a barrel around a central substrate translocation pore [60,61,62] and the N- and C-termini of the proteins are in the mitochondrial intermembrane space [63,64,65].

SLC25A51 is quite different from the other members of the SLC25 family, displaying distinct variations as shown by sequence comparison with typical SLC25 representatives (see Figure 2). Most noteworthy, the signature motif lacks the first negatively charged residue in the first and third repeat (exhibiting a glutamine and an asparagine, respectively), the first positively charged residue in the first and second repeats (showing a leucine and a glutamine, respectively), as well as the first glycine in the third repeat (having a lysine) (Figure 2). In the conformational changes of MCs occurring during the translocation of the substrates, the first two charged residues of each signature motif form an interconnected charged network of salt bridges between H1, H3, and H5 that take part in the closing of the substrate translocation pore towards the mitochondrial matrix (i.e., of the matrix gate) [60]. Given that SLC25A51 lacks many of the aforementioned charged residues in the signature motif, it is left with only one salt bridge (between H3 and H5) out of the three characteristic ones of the matrix gate. Other differences of SLC25A51 with respect to the sequence features of a typical MC are: the possibility of only one salt bridge (between H4 and H6) out of the usual three of the cytoplasmic gate (which closes the substrate translocation pore towards the intermembrane space) and the lack of cardiolipin-binding motif 1 (Figure 2) [62]. By contrast, other MC motifs are quite conserved in SLC25A51: the GXXXG motif and other small residues taking part in helical packing, the cardiolipin-binding motif 2 (corresponding to the last three residues of the signature motif), and the glutamine and tyrosine braces participating in the matrix and cytoplasmic gates, respectively (Figure 2) [66].

**Figure 3:**
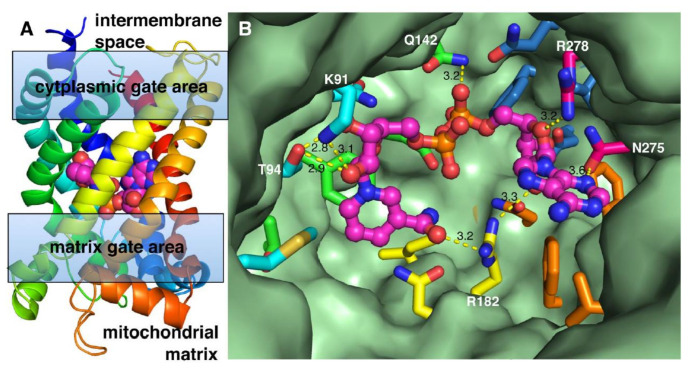
Docking of NAD^+^ into the structural homology model of SLC25A51. A structural homology model of SLC25A51 was generated based on the bovine and yeast ADP/ATP carrier structures (PDB ID: 1OKC, 4C9Q, and 4C9G) by Modeller [68]. Conformationally flexible NAD^+^ was docked into the active site of the SLC25A51 structural model with flexible side chains by Autodock Vina [69]. (**A**) The highest-ranked docking solution (binding energy = −9.9 kcal/mol; interface area = 532 Å^2^) is displayed with the protein in cartoon with rainbow colours from N-terminus (blue) to C-terminus (red) and NAD^+^ in spheres (carbons in magenta) viewed from the mitochondrial inner membrane plane. (**B**) The residues of the substrate binding site of the same docking solution are viewed from the intermembrane space side: NAD^+^ and the residues interacting with it are shown in ball-and-stick and sticks, respectively, with carbons in the same colour as in (**A**).

Based on available structures of the ADP/ATP carrier [60,61], which have been used to understand substrate binding and the translocation mechanism of MCs, a 3D-model of SLC25A51 was generated in a conformation with the matrix gate closed and the cytoplasmic gate open (Figure 3). The substrate binding site of MCs is mainly formed by the so-called “contact point” residues (indicated in yellow in Figure 2), which are located in the even-numbered transmembrane helices and protrude in the substrate translocation pore approximately at the midpoint of the membrane between the matrix and cytoplasmic gates (four, two, and one residues on H2, H4, and H6, respectively, Figure 3) [67]. NAD^+^ was docked into the SLC25A51 homology model to get structural insight into protein-substrate interactions (Figure 3). In the highest-rated docking solution, NAD^+^ is bound in the central cavity of the SLC25A51 structural model at the midpoint between the intermembrane space and the matrix sides of the membrane, and between the cytoplasmic and matrix gate areas (Figure 3A). It is noteworthy that the substrate interacts with residues of all six transmembrane helices (Figure 3B) and forms a total of nine hydrogen bonds with the carrier. In particular, NAD^+^ is bound through hydrogen bonds with each of the three contact points interacting with six out of the seven contact point residues. Of course, the hypothetical substrate binding site of SLC25A51, suggested by this docking solution, awaits experimental validation. However, it is worth mentioning that in most of the other lower-ranking docking solutions, NAD^+^, although having different conformations and interacting with different residues, was bound at the midpoint of the membrane through multiple hydrogen bonds with contact point residues and other residues of all the transmembrane helices. Most likely, this is a consequence of NAD^+^ being one of the largest substrates known for mitochondrial carriers and having many potential hydrogen bond donors and acceptors.

## 7. Hypotheses about the Evolution of SLC25A51

**Figure 4:**
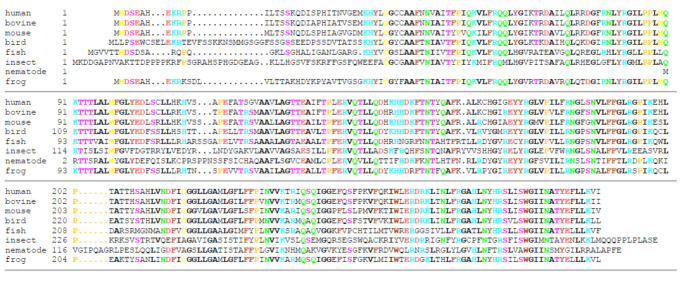
Multiple sequence alignment of representative SLC25A51 homologs from various species: human (*Homo sapiens*, NP_219480), bovine (*Bos taurus*, NP_001092864), mouse (*Mus musculus*, NP_001009949), bird (*Gallus gallus*, XP_003643156), fish (*Danio rerio*, XP_002664343), insect (*Drosophila melanogaster*, NP_651766), nematode (*Caenorhabditis elegans*, NP_501642), and frog (*Xenopus tropicalis*, NP_001107506). The conserved residues are coloured based on the property of the side chain: hydroxyl group S and T (magenta); amide group N and Q (green); positively charged R, K, and H (cyan); negatively charged D and E (red); aromatic F, W, and Y (brown); sulfur-containing C and M (light yellow); hydrophobic A, G, L, I, and V (black and bold); P (dark yellow).

**Figure 5:**
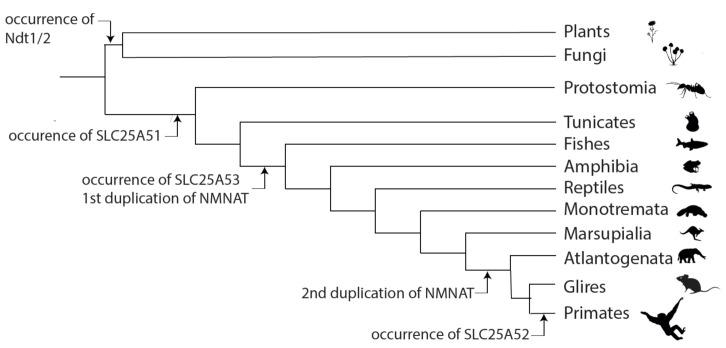
Evolutionary events related to compartmentation of NAD^+^ biosynthesis. The scheme illustrates major evolutionary events related to mitochondrial NAD^+^ metabolism. Ndt1/2 can only be found in plants and fungi, whereas SLC25A51 homologues can be detected in all Metazoan starting from Protostomia. The functionally uncharacterized paralogue SLC25A53 is first found in vertebrates, whereas SLC25A52 can only be found in primates. In parallel, the different isoforms of NMNATs developed. The first duplication of NMNATs in the last common ancestor of vertebrates gave rise to NMNAT2, whereas the later gene duplication in mammals lead to the development of the mitochondrial isoform NMNAT3 [75]. The tree is a schematic representation of selected taxa and is based on information from Tree of Life Web Project [76].

The *SLC25A51* gene is conserved with at least 45% sequence identity on protein level in mammals, birds, fish, amphibians, insects, and nematodes (Figure 4). Notably, it is not found in plants or fungi, where other MCs have been found to transport NAD^+^ [10,11,12,13,14]. Human SLC25A51 is a close paralog of SLC25A52 (Figure 2, sharing 96% identical amino acid sequence, also called MCART2) and, to a lesser extent, to SLC25A53 (30% sequence identity, MCART6) [15,16,17]. These three MCs are found on the same branch in phylogenetic trees and are distinct from all other human SLC25 members [18,70,71]. They are also distant from all other MCs known to transport NAD^+^ (less than 18% overall sequence identity): the human peroxisomal SLC25A17 [13]; yeast mitochondrial Ndt1p and Ndt2p [10]; *Arabidopsis thaliana* AtNDT1 and AtNDT2 [11] and peroxisomal PXN [12,14]. Differently from these NAD^+^-transporting carriers, SLC25A51, SLC25A52, and SLC25A53 do not have a tryptophan, instead of the first negatively charged residue in the second signature motif (Figure 2). SLC25A33 and SLC25A36 have this tryptophan too, although they transport pyrimidine nucleotides and not NAD^+^ [50]. Furthermore, the residues of the contact points and the conserved three-fold-symmetry-related positions typical for NAD^+^-transporting MCs are different from those of SLC25A51, which are almost identical to those of SLC25A52 and SLC25A53. Moreover, it is noteworthy that the genes of human SLC25A51, SLC25A52, and SLC25A53 do not have introns, although homologues in lower species do. SLC25A51 first appeared in Protostomia and is related to oxaloacetate-type SLC25 transporters [72]. SLC25A53 appears to have evolved from SLC25A51 in vertebrates, whereas SLC25A52 can only be found in primates (schematic overview Figure 5). Plant and fungal Ndt1/2, on the other hand, appear to have evolved independently and are more closely related to the mitochondrial folate and FAD transporters [73,74]. In addition, our analyses indicate that SLC25A51 binds NAD^+^ in a different way than the yeast and plant MCs transporting NAD^+^ given that their sequences and the residues of their substrate binding sites are so different.

## 8. Conclusions and Perspectives

The identification of SLC25A51 as a human mitochondrial NAD^+^ carrier is a leap forward in understanding cellular NAD^+^ metabolism and how NAD^+^ pools are distributed between the cytoplasm and mitochondria. It strengthens the hypothesis that NAD^+^ biosynthesis does not necessarily occur in mitochondria. Just as its counterparts in yeast and plants, as well as the peroxisomal NAD^+^ carriers, the mitochondrial NAD^+^ transporter of animals—SLC25A51—is a member of the mitochondrial carrier SLC25 family. However, it seems to have a distant evolutionary origin and a different substrate binding site with respect to the other NAD^+^ carriers. The appearance of SLC25A51 in Protostomia may reflect changes or differences in cellular NAD^+^ utilization, metabolism, and regulation compared to lower organisms or to yeast and plants.

On the other hand, the discovery of a human mitochondrial NAD^+^ carrier SLC25A51 raises a number of important questions, which need to be addressed in future research. What is the role of NMNAT3 in mitochondria? Is NMNAT3 involved in regulation of the mitochondrial NAD^+^ pool, in the synthesis or degradation of NAD^+^ from or to NMN in certain cells or conditions? Does SLC25A51 play a role in the concentration gradient of NAD^+^ between mitochondria and cytoplasm (higher in mitochondria) of various tissues and cell types? The transport properties of SLC25A51 have not been defined yet. What is the substrate specificity of SLC25A51? What is its ability to transport putative substrates such as NAD^+^, NMN, NAAD, α-NAD^+^, NADH, NAMN, NADP, NADPH, Nam, NA, FAD, FMN, and (deoxy-) nucleotides? Does SLC25A51 catalyse unidirectional transport and/or antiport of substrates, and what are its kinetic parameters? A biochemical characterization of SLC25A51 to answer the last two questions would help to understand what the physiological substrates of this carrier protein are and the preferred direction. An additional area of interest for future studies is in elucidating how this transporter is regulated in vivo, for example, by post-translational modifications. Regulation could be critical, because high expression of the carrier or a functional homolog affects mitochondrial function. A further issue is that SLC25A51 does not appear to be essential, suggesting that there may be other mitochondrial transporters capable of transporting NAD. The very close paralog SLC25A52 is likely to have similar transport properties as SLC25A51, but there could be differences.
